# The role of gut microbiota in diarrhea and its alleviation through microbiota-targeted interventions

**DOI:** 10.3389/fmicb.2025.1630823

**Published:** 2025-10-28

**Authors:** Rui Tian, Chu-Jun Chong, Ya-Ya Bai, Ni Chen, Rui-Rui Qiao, Kan Wang, Yu-Wei Wang, Peng Zhao, Chong-Bo Zhao, Yu-Ping Tang, Li Zhang, Qiao Zhang

**Affiliations:** ^1^Key Laboratory of Shaanxi Administration of Traditional Chinese Medicine for TCM Compatibility, and State Key Laboratory of Research & Development of Characteristic Qin Medicine Resources (Cultivation), and Shaanxi Collaborative Innovation Center of Chinese Medicinal Resources Industrialization, and Shaanxi Traditional Chinese Medicine Processing Technology Heritage Base, Shaanxi University of Chinese Medicine, Xi’an, China; ^2^College of Sports and Health, Nanjing Sport Institute, Nanjing, Jiangsu, China; ^3^Hanlin College Nanjing University of Chinese Medicine, Taizhou, Jiangsu, China

**Keywords:** diarrhea, gut microbiota, metabolites, probiotics, fecal microbiota transplantation, bacteriophage therapy

## Abstract

Diarrhea is a common gastrointestinal disease and closely related to the balance of the gut microbiota (GM). In turn, dysregulation of the GM can affect the onset and progression of diarrhea through regulating the metabolism, intestinal immune function, intestinal barrier function and changes in the brain-gut axis of host. Although increasing evidence suggests that GM is associated with gastrointestinal homeostasis and disease, the underlying mechanisms are not fully understood. GM disorder was often accompanied by diarrhea patients and animals, and the diarrhea caused by GM imbalance mainly involved the effects on short chain fatty acids (SCFAs), bile acids (BAs), intestinal barrier, immune system, and brain-gut microbiota axis (BGMA). In addition, intervening in the GM (probiotics, fecal microbiota transplantation and bacteriophage therapy) has been shown to be an effective way to alleviate diarrhea. In this review, the mechanism of diarrhea occurrence, probiotics, fecal microbiota transplantation and bacteriophage therapy intervene in diarrhea by regulating GM from basic and clinical research were summarized and discussed. We aim to provide the latest reference for studying the mechanism of treating diarrhea from the perspective of GM, and provide data support for clinical treatment of diarrhea.

## 1 Introduction

Despite improvements in living conditions and widespread vaccination, diarrhea remains one of the most prevalent global health issues, resulting in approximately 1.3 million deaths annually (Collaborators G.D.D, 2017; Stockmann et al., 2017). Diarrhea is an intestinal disorder characterized by increased gastrointestinal motility, leading to elevated stool frequency and higher water content, often presenting as watery stools. It can be caused by a variety of pathogens and other factors (Brehm et al., 2020; Schiller et al., 2017). Common forms of diarrhea include infectious diarrhea (caused by bacteria, viruses, parasites, or fungi), organic-associated diarrhea, antibiotic-associated diarrhea (AAD), functional diarrhea, and diarrhea-predominant irritable bowel syndrome (IBS-D) (Wilkins and Sequoia, 2017). Although the current pharmaceuticals for diarrhea includes various agents from traditional Chinese (e.g., Shen-Ling-Bai-Zhu-San) and Western medicine (e.g., loperamide, diphenoxylate), the development of novel, safer, and more effective treatment strategies is urgently required (Ali et al., 2020; Khan et al., 2019; Wang et al., 2020).

The gut microbiota (GM) is increasingly recognized as a pivotal factor in human health, influencing nutrient absorption, immune regulation and gastrointestinal homeostasis (Paul et al., 2025). Alterations in the composition and function of the GM, often termed GM dysregulation, are closely linked to the development of gastrointestinal diseases (Quaglio et al., 2022). The onset of diarrhea is frequently accompanied by GM disturbances, aberrant metabolite levels, reduced immune function, and impaired intestinal barrier function (Anbazhagan et al., 2018; Wu et al., 2022).

For instance, patients with IBS-D exhibit an increased relative abundance of *Shigella, Enterococcus, Streptococcus* and *Ruminococcus,* alongside a decreased abundance of *Faecalibacterium* (Wei et al., 2020). Notably, *Faecalibacterium* is a dominant butyric acid-producing genus. Butyric acid serves as a crucial energy source for colonocytes and can exert anti-inflammatory, immunomodulatory, and intestinal barrier-protecting functions by inhibiting the activation of the toll-like receptor 4-myeloid differentiation factor 88-nuclear factor-*κ*B (TLR4-MyD88-NF-*κ*B) signaling pathway. However, a decline in *Faecalibacterium* abundance is frequently associated with diarrheal symptoms and intestinal inflammation (Anbazhagan et al., 2024; Karim et al., 2024). Moreover, it was found that the occurrence of IBS-D was closely related to the expression of 5-Hydroxytryptamine (5-HT) in the brain-gut microbiota axis (BGMA), and *Ruminococcus* can regulate the production of 5-HT through Trace Amine-Associated Receptor 1 (TAAR1) signaling mediated by phenethylamine and tryptamine, which can stimulate gastrointestinal transit and lead to diarrhea in patients with IBS-D (Shen et al., 2022; Zhai et al., 2023). Studies in AAD models have shown reduced GM richness and diversity, downregulation of the tight junction (TJ) protein zonula occluden 1 (ZO-1) in the colon, and elevated levels of pro-inflammatory cytokines including interleukin-2 (IL-2), interleukin-1β (IL-1β), and tumor necrosis factor-*α* (TNF-α) (Cui et al., 2021; Xu H. et al., 2023). Collectively, these findings highlight the pivotal role of GM in the pathogenesis of diarrhea (Gallardo et al., 2020; Mei et al., 2021).

Recently, probiotic interventions, fecal microbiota transplantation (FMT) techniques and bacteriophage therapy have demonstrated considerable potential in the treatment of diarrhea (Fujimoto and Uematsu, 2022; Lai et al., 2019; Liu M. et al., 2024). *Lactiplantibacillus plantarum* P9 has been shown to alleviate diarrhea by regulating the composition of GM and increasing the patient’s functional intestinal metabolites. The specific action mechanisms include increased the relative abundances of *Butyricicoccus_A* sp002395695 and *Streptococcus thermophilus*, reduced the relative abundances of *Phascolarctobacterium faecium* and *Faecalibacterium* sp., increased the content of acetic acid and butyric acid in short chain fatty acids (SCFAs), and decreased the level of deoxycholic acid (Yang et al., 2024). Additionally, studies have reported that transplantation of fecal fluid from healthy donors into AAD patients alleviated GM disorders, modulated GM composition and quantity, and lowered interleukin-8 (IL-8) and C-reactive protein (CRP). These changes thereby enhanced intestinal immune function and alleviated AAD symptoms (Wang L. et al., 2024). Furthermore, the study found that phage A221 effectively treated diarrhea caused by *Escherichia coli* (*E. coli*) GXXW-1103 in weaned piglets, increased their daily weight gain, and reduced the proportion of Enterobacteriaceae in the duodenum to 0.64%, thereby alleviating lesions in the cecum and duodenum (Mao et al., 2023). Thus, targeted modulation of the GM represents a promising therapeutic strategy for alleviating diarrhea.

Therefore, in this review, we summarize the regulatory mechanisms of GM and its metabolites in diarrhea. Specifically, we discuss the mechanisms of probiotics, FMT and bacteriophage in the treatment of diarrhea, aiming to provide insights for future research on targeted modulation of the GM as a therapeutic strategy for diarrheal diseases.

## 2 The pivotal role of GM in diarrhea related diseases

As a complex and diverse ecosystem, the GM colonizes the entire gastrointestinal tract in a symbiotic fashion, participating in the growth and development of the host organism while regulating the body’s immune system (Takiishi et al., 2017). The GM is crucial for maintaining host homeostasis and overall health. Its diversity and abundance have direct implications for disease pathogenesis and clinical treatment outcomes (Chen et al., 2021). Under normal physiological conditions, the species composition and proportional distribution of GM remain in a homeostatic balance, and it exerts multiple pivotal functions in the host, including modulating immune responses, mediating metabolic processes, and sustaining the homeostasis of the intestinal barrier (Jandhyala et al., 2015; Wu and Wu, 2012). In contrast, under pathological circumstances, perturbations to the intestinal microecosystem can disrupt this balance, ultimately resulting in GM dysbiosis. This dysbiosis subsequently impairs host health via a variety of mechanisms, including alterations in SCFAs metabolism (Morrison and Preston, 2016), BAs (Cai et al., 2022), intestinal barriers (Allam-Ndoul et al., 2020), the immune system (Donald and Finlay, 2023), and BGMA (Hillestad et al., 2022). Such alterations may contribute to the development of diseases such as diarrhea (Shao et al., 2020), inflammatory bowel disease (Quaglio et al., 2022), and cardiovascular diseases (Witkowski et al., 2020).

### 2.1 Effect on the composition of the GM

Under physiological conditions, a homeostatic GM supports key host functions including immune regulation, metabolic processes, and the maintenance of intestinal barrier integrity (Yue et al., 2019).

In healthy individuals, the GM is predominantly composed of the phyla Firmicutes and Bacteroidetes, followed by Actinobacteria and Verrucomicrobia (Hollister et al., 2014). Under normal physiological conditions, GM is in homeostasis and plays functions of immunity, metabolism and maintenance of intestinal barrier homeostasis in the body (Yue et al., 2019). However, the composition and diversity of GM is easily influenced by various factors (such as diet, drugs, pathogens, and environmental factors), further affecting human and animal health (Cryan et al., 2019; Lange et al., 2016; Zhang, 2022). Compelling evidence indicates that GM dysbiosis increases host vulnerability to a broad spectrum of pathogens and promotes the development of diverse diseases, including diarrhea, IBS, and allergies (Leong et al., 2018; Shchikota et al., 2021).

Despite improvements in living conditions and healthcare in recent years, diarrhea remains a globally prevalent issue (Wolf et al., 2022). A topic of growing interest currently is the interplay between diarrhea and the GM, which is featured by pathogen-dominated GM dysregulation, encompassing bacterial, fungal, and viral etiologies (Li et al., 2021). Invasive pathogens suppress the proliferation of commensal bacteria, thereby reducing beneficial gut microbiota and increasing pathogenic strains. This imbalance further induces intestinal dysfunction and activates immune responses, ultimately culminating in diarrhea (Czepiel et al., 2019; Jesser et al., 2023).

Furthermore, it has been observed that other types of diarrhea are also closely associated with alterations in the GM (Table 1). Thus, it is evident that dysbiosis of the GM exists across various forms of diarrhea.

**Table 1:** Changes of GM in diarrhea.

Type of diarrhea		Classification level	Changes in microbiota	Reference
Infectious diarrhea	Bacterial diarrhea (*E. coli* O_101_)	Phylum level	Proteobacteria and Actinobacteria increased; Firmicutes and Verrucomicrobia decreased.	Sun et al. (2019)
		Genus level	*Escherichia* and *Shigella* increased; *Prevotella*, *Enterococcus* and *Akkermansia* decreased.	
	Virus diarrhea (Rotavirus)	Genus level	*Acinetobacter* increased; Ruminococcaceae NK4A214 decreased.	Mizutani et al. (2021)
	Virus diarrhea (Norovirus)	Genus level	*Holdemanella*, *Staphylococcus*, *Howardella*, *Corynebacterium*, and *Massilia* increased.	
	Parasitic diarrhea (Giardia)	Phylum level	Proteobacteria increased, Firmicutes and Melainabacteria decreased.	Barash et al. (2017)
Organic - associated diarrhea	Post-cholecystectomy diarrhea	Phylum level	Bacteroidota increased, Firmicutes decreased.	Xu Y. et al. (2022)
		Genus level	*Prevotella* and *Enterococcus* increased, *Alistipes*, *Lactobacillus*, *Ruminococcus* and *Bacteroides* decreased.	
Antibiotic - associated diarrhea (AAD)		Phylum level	Proteobacteria increased, and Firmicutes, Bacteroidetes, Actinobacteria and Planctomycetes decreased.	Shao et al. (2020)
		Genus level	*Enterococcus* and *Clostridium* increased, *Lactobacillus* decreased.	
Functional diarrhea		Phylum level	*Bacteroides* increased, Firmicutes and Proteobacteria decreased.	Li X. T. et al. (2023)
		Genus level	*Akkermansia* increased, *Lactobacillus*, *Ruminococcus* and *Allobaculum* decreased.	
IBS-D		Phylum level	Proteobacteria increased, Firmicutes, Fusobacteria, and Actinobacteria decreased.	Mei et al. (2021)
		Genus level	Enterobacteriaceae increased, *Alloprevotella* and *Fusobacterium* decreased.	

### 2.2 Effect on metabolites of the GM

SCFAs, including acetate, propionate, and butyrate (Liu et al., 2021), are mainly produced by GM via anaerobic fermentation of indigestible carbohydrates and host-derived substrates (Zhang et al., 2023). SCFAs contribute to the enhancement of intestinal barrier function, exhibit anti-inflammatory effects, and participate in immunomodulation (Parada Venegas et al., 2019). Specifically, acetate and propionate serve as energy sources for peripheral tissues (den Besten et al., 2013). A recent study demonstrated that acetic acid mediates crosstalk between epithelial and immune cells and promotes T cell-dependent immunoglobulin A (IgA) production by stimulating CD4^+^ T cells (Takeuchi et al., 2021). Moreover, propionate has been shown to prevent the reduction of TJ proteins, such as ZO-1 and occludin, in colon tissue, and to suppress the mRNA expression of pro-inflammatory cytokines IL-1β, IL-6, and TNF-*α* (Tong et al., 2016). Butyrate also exerts anti-inflammatory effects by inhibiting the secretion of IL-8, IL-6, IL-12, and TNF-α, while promoting the production of the anti-inflammatory cytokine IL-10, thereby contributing to the maintenance of the intestinal epithelial barrier (Lee et al., 2017). Additionally, they promote the growth of beneficial bacteria, improve GM composition, and help regulate host immune homeostasis (Fukuda et al., 2011; Mann et al., 2024). Moreover, a separate study demonstrated that propionic acid, secreted by *Akkermansia muciniphila*, binds to G-protein-coupled receptor 43 on the surface of intestinal epithelial cells. This interaction enhances histone acetylation, which in turn upregulates the expression of TJ proteins occludin and ZO-1 and increases mucin levels, ultimately improving the integrity of the intestinal epithelial barrier (He et al., 2023). In addition, another study demonstrated that, relative to healthy calves, calves with diarrhea induced by bovine rotavirus (BRV) exhibited significantly reduced concentrations of total SCFAs, acetic acid, propionic acid, and isocaproic acid; in contrast, only propionic acid concentrations were markedly decreased in calves with diarrhea caused by bovine coronavirus. Notably, the depletion of *Parabacteroides* and *Ruminococcus* was strongly associated with reduced acetic acid levels, while declines in isocaproic acid content were closely linked to the loss of *Parabacteroides*, *Ruminococcus*, *Fournierella*, and *Rikenellaceae_RC9_gut_group*. Furthermore, a significant reduction in propionic acid concentrations showed a positive correlation with the depletion of *Collinsella* (Cui et al., 2023). Both propionic and butyric acids are believed to enhance the integrity of epithelial cells, with butyric acid serving as the primary energy source for colonocytes (Furusawa et al., 2013; Tong et al., 2016). Reduced butyrate levels have been shown to elevate intestinal oxygenation, which not only drives gut microbial dysbiosis and promotes the expansion of aerobic pathogens but also disrupts intestinal homeostasis (Handa et al., 2023). SCFAs are absorbed by epithelial cells, which in turn stimulates Na^+^-dependent absorption of water and electrolytes, thereby mitigating diarrhea symptoms (Binder, 2010). Several studies have indicated that ADD-type mice exhibit reduced levels of SCFAs (Min et al., 2024; Yang L. et al., 2021; Zhan et al., 2023). Furthermore, piglets infected with *E. coli* developed diarrhea and exhibited decreased levels of SCFAs in their feces (Liu et al., 2019).

Bile acids (BAs) serve as essential signaling molecules that significantly regulate glucose homeostasis, lipid metabolism, and energy expenditure (Sah et al., 2022; Yu et al., 2023). They consist of primary bile acids (PBAs), which are synthesized by the liver, and secondary bile acids (SBAs), which are metabolized by the GM. Among them, PBAs include chenodeoxycholic acid (CDCA) and cholic acid (CA), while SBAs comprise lithocholic acid (LCA) and deoxycholic acid (DCA). Nearly 95% of luminal BAs are reabsorbed in the distal ileum, while the remainder undergoes microbial modification by the GM prior to excretion or passive absorption. In humans, the GM is instrumental in the generation of SBAs via a series of enzymatic reactions, including deconjugation, 7*α*-dehydroxylation, oxidation, epimerization, desulfation, and esterification. Of these, deconjugation and 7α-dehydroxylation are the most physiologically significant processes (Ridlon et al., 2016; Wahlström et al., 2017). When intestinal homeostasis is disrupted, dysbiosis of the GM affects BAs metabolism and ultimately alters the host response. In IBS-D, excessive fecal BAs are considered a contributing factor to pathogenesis, and there are higher levels of PBAs in fecal samples of IBS-D patients compared to healthy subjects (Duboc et al., 2012; Wei et al., 2021; Wei et al., 2020). However, research has indicated that a microbiota rich in Clostridia can promote BAs excretion in IBS-D patients (Zhao et al., 2020). It is well established that BAs modulate intestinal mucosal permeability and participate in inflammatory responses. Specifically, CDCA and DCA exert their effects by promoting epidermal growth factor receptor (EGFR) autophosphorylation and occludin dephosphorylation, leading to the reorganization of occludin within TJs and a consequent increase in paracellular permeability (Raimondi et al., 2008). Additionally, CDCA contributes to pro-inflammatory responses by stimulating the release of IL-8 and reactive oxygen species (ROS), as well as amplifying the effects of TNF-α and IL-1β on interferon-*γ* (IFN-γ) production (Sarathy et al., 2017). Ursodeoxycholic Acid (UDCA) has been shown to reduce the production of inflammatory cytokines by participating in the BA receptor Farnesoid X Receptor (FXR), while inhibiting NF-*κ*B activation in macrophages (Pi et al., 2023). BAs metabolites were found to be excessive in the feces of Primary Sclerosing Cholangitis (PCD) patients and PCD mice, and SBAs [DCA, LCA and Hyodeoxycholic Acid (HDCA)] were found to be associated with the onset of diarrhea. These SBAs shortened the gastrointestinal transit time by 0.6-fold, increased the fecal water content by 1.3-fold and stimulated 5-HT levels *in vitro* and *in vivo* (Xu Y. et al., 2023). However, blocking BAs conjugated Takeda G protein-coupled receptor 5/Transient Receptor Potential Ankyrin 1 (TGR5/TRPA1) receptors significantly alleviated PCD GM-induced diarrhea. The present study demonstrates that GM and BA metabolism play a role in diarrhea. These results offer promising biomarkers for diagnosing and treating diarrhea and lay the groundwork for further investigation.

Branched chain amino acids (BCAAs), which include leucine (Leu), isoleucine (Ile), and valine (Val), are essential amino acids for the human body (Peng et al., 2020). These amino acids exert direct or indirect effects on diverse physiological functions, including energy metabolism, protein synthesis, and immune responses (Ma et al., 2018; Stipanuk, 2007). Similarly, BCAAs function as modulators that promote intestinal development and enhance gut health (Ren et al., 2016; Ren et al., 2015). Currently, the majority of research has concentrated on the function of Leu, with less attention paid to Val and Ile in the gut. Leu can maintain intestinal health by enhancing TJ in fish (Jiang et al., 2015). Additionally, it has been shown to improve intestinal epithelial cell proliferation, increase villus height, and promote growth in the small intestine of pigs. However, intestinal growth was inhibited when Leu levels were as high as 2.57% (Ren et al., 2015). Dietary Ile improves intestinal immune function and microbial population, and regulates gene expression of antioxidant enzymes, TJ, Nuclear factor erythroid 2-related factor 2 (Nrf2), Kelch-like ECH-associated protein 1 (Keap1), p38, and Extracellular Signal-regulated Kinase 1 (ERK1) in the intestine of Jian carp (Zhao et al., 2014). Additionally, BCAAs have been significantly linked to diarrhea; a decreasing trend in BCAA levels was observed in both the functional diarrhea group and the IBS-D group (James et al., 2023). Rotavirus infection induces diarrhea in weaned pigs via systemic protein metabolic disorders and jejunal mucosal dysfunction. However, dietary supplementation with 1% leucine alleviated rotavirus-induced diarrhea in weaned pigs, potentially due to leucine’s roles in enhancing protein metabolism, improving intestinal digestive and absorptive capacities, and reinforcing the non-specific barrier function of the intestinal mucosa (Mao et al., 2015). Furthermore, L-isoleucine supplementation has been found to significantly reduce stool output and fluid intake in children suffering from non-cholera acute watery diarrhea (Alam et al., 2011). More recently, a study demonstrated that supplementation with Ile increased the relative abundance of *Prevotella* and decreased the relative abundance of Rikenellaceae in the colon of diarrhea piglets infected with rotavirus, increased the secretion of interleukin-4 (IL-4), IL-10, and Secretory Immunoglobulin A (sIgA), and increased the expression of Claudin-3, Occludin, ZO-1 and mucin 1 (MUC-1), improved the immunity, colon barrier function and colon GM of piglets with diarrhea (Jiang C. et al., 2024; Jiang C. Y. et al., 2024). Nevertheless, the existing literature displays a striking imbalance toward Leu, leaving the mechanisms and efficacy of Val and Ile underexplored. Future studies should thus prioritize elucidating the individualized and synergistic contributions of all three BCAAs—particularly Val and Ile—ac different physiological and pathological contexts, to enable more precise and effective nutritional strategies for intestinal health.

### 2.3 Effect on intestinal barrier function

The intestinal barrier is a complex physiological structure that serves as a physical, biological, chemical, and immunological barrier. It interacts with the external environment and regulates host health (Zhou et al., 2024). The intestinal barrier, being semi-permeable, serves a dual function: it safeguards the internal milieu against the potential translocation of pathological molecules and microorganisms, while facilitating the absorption of nutrients and water (Martini et al., 2017). However, in pathological conditions, the integrity of the intestinal barrier can be compromised, leading to many local and systemic diseases (Aleman et al., 2023; Wang et al., 2022). TJs serve as a crucial form of connection between intestinal epithelial cells.

The proteins ZO-1 and occludin are key structural components of TJs. They are essential for maintaining cellular morphology and TJ structural integrity, and are widely used as indicators for assessing intestinal barrier function (Al-Sadi et al., 2011; Haas et al., 2022). Mucins are the primary glycoproteins that constitute the intestinal mucosal barrier. Among them, Mucin 2 (MUC2) is the most secreted mucin in the gastrointestinal tract and maintains the integrity of the mucus barrier, which is closely related to GM homeostasis (Liu et al., 2023; Yao et al., 2021). Tropini et al. demonstrated that diarrhea is closely related to the GM and the intestinal mucus barrier (Tropini et al., 2018). Diarrhea significantly disrupts the GM and is associated with thinning or loss of the intestinal mucus layer. This effect may be linked to dysregulated expression of the tight junction proteins ZO-1 and occludin, which compromises intestinal barrier integrity and increases permeability (Chen H. R. et al., 2024; Tropini et al., 2018).

Impaired intestinal mucosal barrier function serves as the primary pathological basis for the development of IBS-D (Shi et al., 2023). Upon the onset of IBS-D, patients exhibit a significant reduction in the expression of occludin, ZO-1, and other epithelial tight junction proteins, resulting in compromised intestinal epithelial barrier integrity and elevated intestinal permeability (Guo et al., 2023; Wang L. et al., 2023). Recent studies have found that *Lactobacillus* promotes occludin and ZO-1 expression and improves diarrheal symptoms (Hou et al., 2020). Related studies have further demonstrated that in diarrheic piglets infected with *E. coli*, increased abundances of *Lactobacillus* and *Cyanobacterium* are associated with reduced intestinal permeability and enhanced barrier repair, with *Lactobacillus* showing a particularly strong correlation with key intestinal barrier markers (Luo et al., 2022). Xu et al. found that MUC2 is a crucial protein in the prevention and treatment of rotavirus infections and diarrhea. It functions by safeguarding the epithelial barrier and enhancing intestinal permeability resistance (Xu et al., 2016). Wang et al. further observed that elevated MUC2 content in the ileum of diarrheal rats enhances intestinal barrier defense and confers intestinal protection (Wang et al., 2019). Furthermore, MUC2 concentration was significantly lower in AAD mice than in normal mice. A significant negative correlation was also identified between MUC2 and two gut microbial taxa, *Prevotellaceae_NK3B31_group* and *Rothia* (Li C. et al., 2023; Li X. T. et al., 2023). Collectively, these findings demonstrate a close association between diarrhea development, GM composition, and intestinal barrier function.

### 2.4 Effects on intestinal immune function

The GM intricately interacts with the host immune system. The crosstalk between the GM and enterocytes plays a crucial role in shaping the intestinal environment, thereby profoundly influencing intestinal immune homeostasis (Hold, 2016). Different types of diarrhea induce alterations in GM composition, which in turn modulates the expression of inflammatory factors. For example, in patients with diarrhea-predominant IBS-D, levels of IL-8 and TNF-*α* are elevated, while IL-10 is reduced; in mice with AAD, GM dysbiosis is observed, characterized by a marked increase in Proteobacteria and decreases in Bacteroidetes and Firmicutes, accompanied by upregulated IL-1 and IL-6 levels (Chen et al., 2022; Zhen et al., 2015; Zhu et al., 2022). It was observed that *E. coli* O_1_ caused diarrhea in calves with disturbances in the GM and an increased abundance of Proteobacteria and Clostridiales. This condition was accompanied by a decreased expression of CD4^+^ T and an elevated expression of Cluster of Differentiation 8 Positive T Lymphocyte (CD8^+^ T) and CD11c-positive T lymphocyte (CD11c^+^ T) in the ileum. Additionally, there were reduced serum levels of IgA and Immunoglobulin G (IgG), alongside heightened levels of IL-6 and TNF-α (Chen H. et al., 2023). T helper cell 17 (Th17) contribute to the maintenance of host intestinal immune homeostasis through interleukin-17A (IL-17A)-induced expression of the epithelial polymeric immunoglobulin receptor (Cao et al., 2012). In colonic tissues of IBS-D mice, the Th17/Tregs ratio was found to be significantly altered, characterized by reduced Tregs and IL-10^+^Foxp3^+^T cells alongside increased Th17 cells. Correlation analysis further revealed positive associations between *Ruminococcus_gnavus* with the Th17/Tregs ratio (Zhang M. M. et al., 2024; Zhang Y. et al., 2024; Zhang Z. et al., 2024).

It is thus clear that GM plays an important role in regulating intestinal immune homeostasis during diarrhea.

### 2.5 Effects on BGMA

In recent years, this concept has expanded to include BGMA, prompted by the growing recognition of the gut microbiota’s critical role in human health and disease (Lee et al., 2023). The BGMA acts as a bidirectional communication pathway between the central nervous system (CNS) and the gastrointestinal tract. It mediates interactions involving the CNS, enteric nervous system (ENS), neuroendocrine system, and immune system, with signals being transmitted either directly or indirectly between the CNS and ENS (Arneth, 2018; Grenham et al., 2011; Morais et al., 2021).

Notably, microbial metabolic activity profoundly shapes brain–gut signaling. For example, disruptions in tryptophan metabolism impact the synthesis of serotonin (5-HT), a key intermediate, which can activate brain–gut neural circuits and precipitate diarrheal responses (Morais et al., 2021; Spencer and Hu, 2020). Furthermore, central processes modulate gut function via the hypothalamic–pituitary–adrenal (HPA) axis: psychological stress triggers cortisol release, altering intestinal permeability and compounding gut dysfunction (Chen H. et al., 2023; Chen X. et al., 2023; Chen J. et al., 2023; Morais et al., 2021). Evidence underscores a robust association between functional diarrhea and impairment of the BGMA. Notably higher rates of this condition occur in patients with mental disorders, with GM-CNS crosstalk serving as a potential mediator of this comorbidity (Zhang et al., 2021). The pathogenesis of IBS-D involves multifaceted interactions among brain–gut peptides, immune activation, and microbial composition (Li et al., 2020). For instance, Gao et al. demonstrated that dampening HPA axis activity via CRHR1 downregulation alleviates diarrheal symptoms in IBS models, underscoring the therapeutic relevance of BGMA modulation (Gao et al., 2023). Wu et al. provided further mechanistic insight, identifying correlations between specific microbial genera and neuro-immune markers in IBS-D rats. The genus *Paraprevotella* was positively associated with elevated 5-HT, CRF, and NPY, suggesting its potential role in modulating the HPA axis via serotonergic pathways (Wu et al., 2022). Additionally, microbial metabolites such as SCFAs and 5-HT are implicated in bidirectional BGMA communication, and their aberrant levels have been consistently reported in IBS-D patients (Dinan and Cryan, 2017; Luo et al., 2021). Interventions including probiotic supplementation have shown promise in reducing 5-HT levels and ameliorating IBS-D symptoms, highlighting the translational potential of targeting microbial components (Gu et al., 2022; Wu et al., 2024). Another compelling example comes from Chen et al. reported that alkaline mineral complex (AMC) water improved diarrhea resistance in stressed piglets by rebalancing the HPA axis and enriching beneficial bacteria such as *Lactobacillus helveticus* and *Ruminococcus gnavus*. This reinforces the notion that BGMA-oriented interventions can restore gut homeostasis through multifactorial mechanisms (Chen H. et al., 2023; Chen X. et al., 2023; Chen J. et al., 2023).

Overall, research on the BGMA provides critical insights into the mechanisms underlying diarrhea and reveals promising therapeutic potential. Current evidence suggests that targeting the BGMA—through modulation of microbial metabolites, neuroendocrine pathways, and immune signaling—may alleviate both intestinal and psychiatric symptoms. However, most studies to date remain correlative or reliant on animal models, highlighting a need for causal validation and clinical translation. Future work should integrate multi-omics approaches to elucidate precise molecular targets within the BGMA, ultimately facilitating the development of personalized therapies and bridging the gap between mechanistic discovery and clinical application.

**Figure 1:**
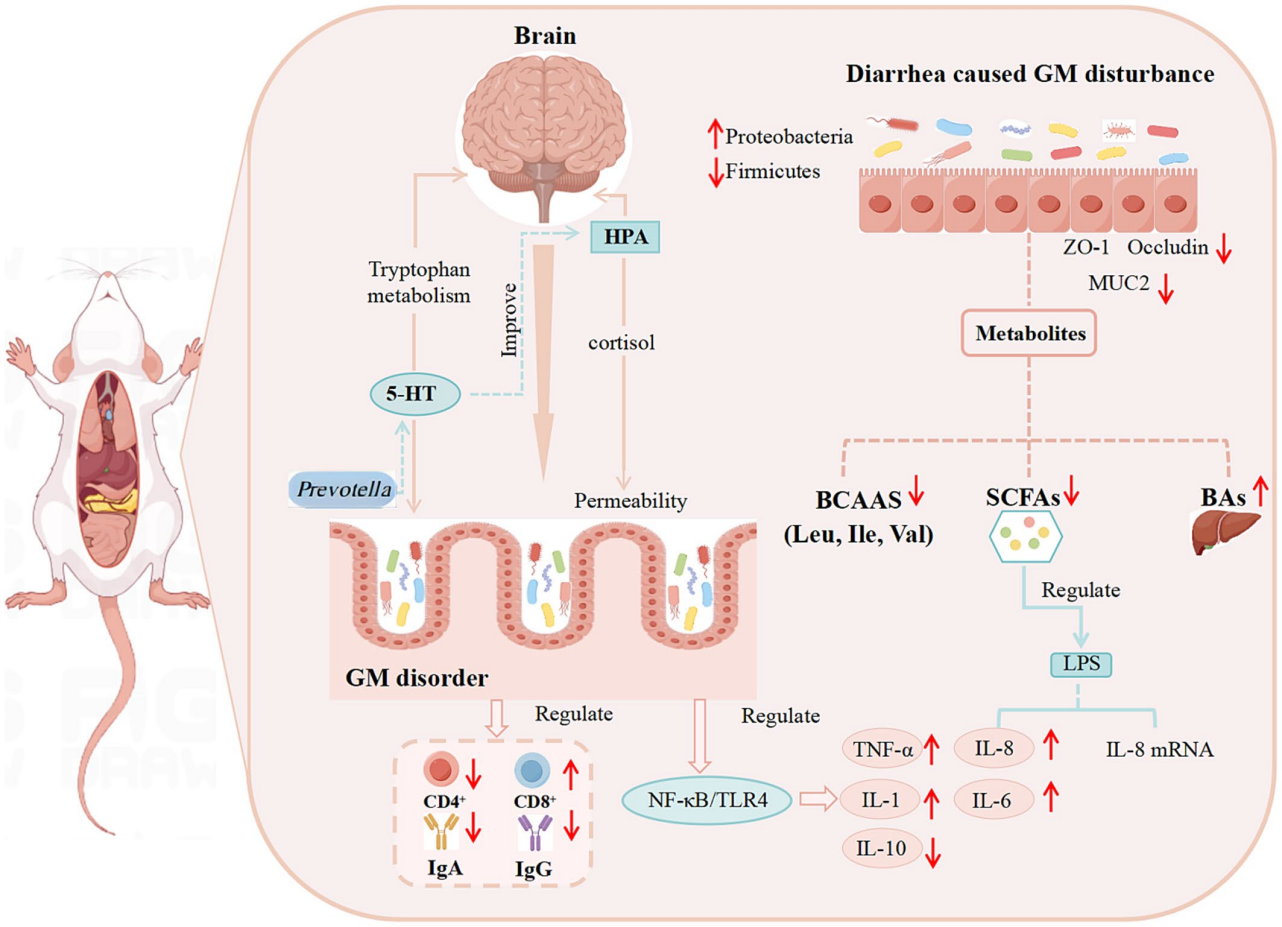
The potential mechanism of diarrhea development. (The relationship between diarrhea and GM, GM metabolites, the immune system, the intestinal barrier, and BGMA).

In summary, the occurrence of diarrhea can alter the composition of the gut microbiota and the levels of its metabolites, regulate immune function, affect the gut-brain axis, and impair intestinal barrier integrity. The potential mechanisms mediating these effects are illustrated in Figure 1.

## 3 Impact of interventions targeting GM on diarrhea

### 3.1 Probiotic interventions

Probiotics are defined as beneficial, viable microorganisms, and a growing body of evidence has shown that numerous probiotic strains alleviate diarrhea by modulating the GM (Wieërs et al., 2019), regulation of inflammatory factor production (Wang F. et al., 2023), and enhancement of the intestinal mucosal barrier function (Camilleri, 2021; Su et al., 2022).

*Lactobacillus* and *Bifidobacterium* are widely employed as probiotics in the treatment of diarrhea, owing to their crucial functions in alleviating inflammation and promoting the balance of the intestinal microbiota. Lactoferricin produced by *Lactobacillus reuteri* CO21 was found to be able to modulate the intestinal physical barrier function by inhibiting the TLR4, Myd88 and Myosin light-chain kinase (MLCK) pathways and up-regulating the expression of the TJ proteins ZO-1 and claudin-2, thereby increasing piglets’ resistance to Enterotoxigenic *E. coli* and alleviating diarrhea (Xie et al., 2021). *Lactobacillus* and *Saccharomyces boulardii* have also shown effectiveness in the prevention or treatment of AAD (Doar and Samuthiram, 2023; Yang Y. et al., 2023; Yang Q. et al., 2023). *Saccharomyces boulardii* alleviates GM disorders and improves intestinal barrier function (Bustos Fernández et al., 2023). *Saccharomyces boulardii* mitigates mucosal injury by modulating intestinal mucin composition and secretion, strengthening the mucin barrier, and reducing SN-38 penetration into epithelial cells (Sezer et al., 2009).

In conclusion, probiotics exert beneficial effects on both the prevention and treatment of diarrhea, with such effects being strain-and dose-dependent. Thus, further studies are required to identify and optimize the selection and application of probiotics for managing different types of diarrhea.

#### 3.1.1 Basic experiments

During animal development, exposure to various pathogenic bacteria and toxic compounds often leads to intestinal barrier dysfunction, thereby contributing to the onset of diarrhea and impaired growth (Kovanda et al., 2023; Satitsri et al., 2016). However, probiotics can modulate intestinal barrier function, alleviate intestinal injury, and mitigate diarrhea.

**Figure 2:**
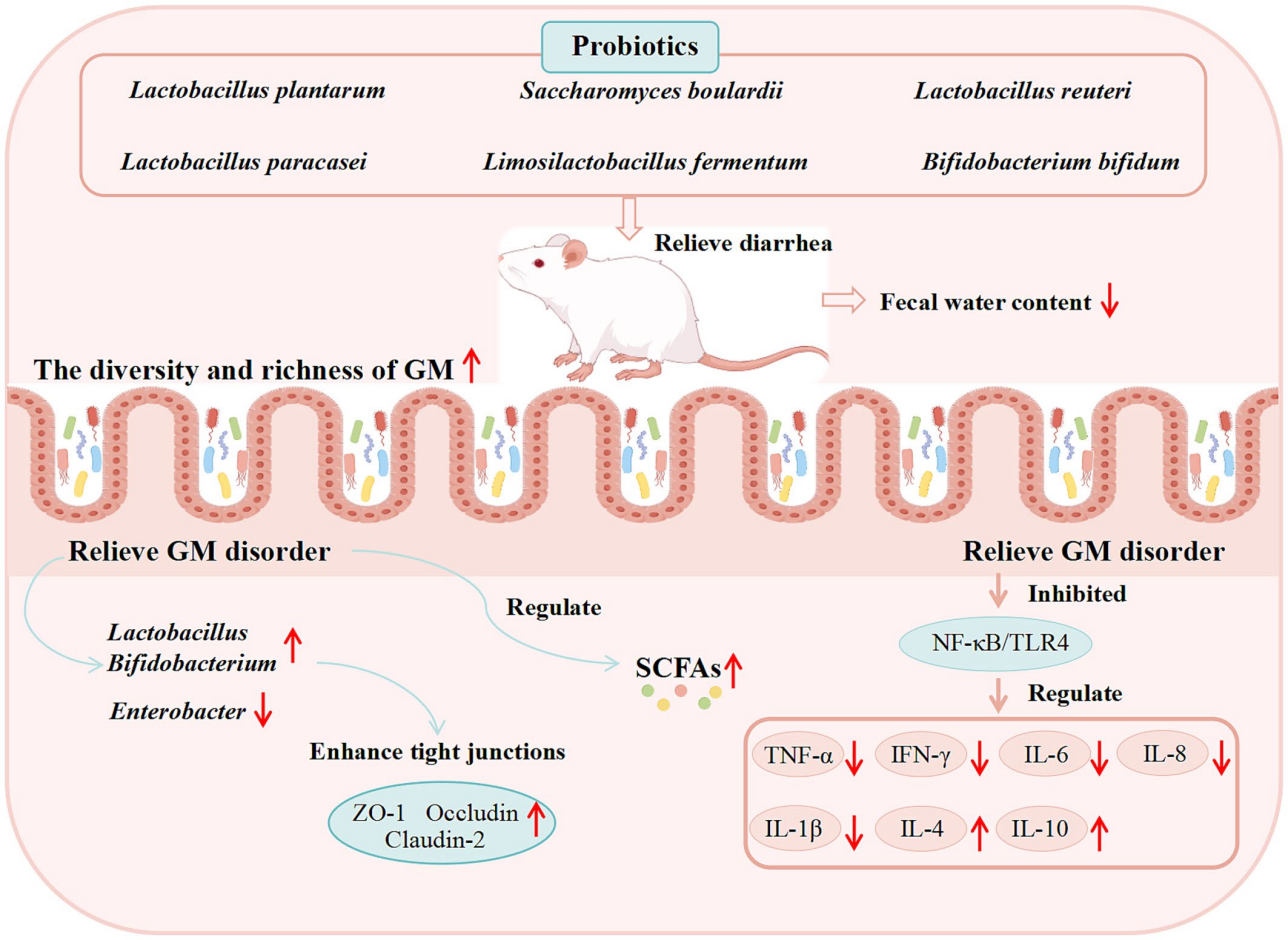
Proposed mechanisms of action of probiotics against diarrhea derived from basic studies. (These potential mechanisms mainly involve the GM, SCFAs, the immune system, and intestinal barrier function).

ETEC is a major pathogen of animal diarrhea (Xu C. et al., 2023; Xu Y. et al., 2023; Xu H. et al., 2023), which disrupts the intestinal epithelial barrier through adhesins and enterotoxins (Zhu et al., 2018). It has been found that *Lactobacillus plantarum* ZLP001 has antimicrobial activity, which prevents ETEC growth by producing certain antimicrobial substances and generating a relatively acidic environment (Wang et al., 2018b). Treatment with *L. plantarum* ZLP001 alleviated ETEC-induced intestinal damage by preserving the expression of TJ proteins (claudin-1, occludin, ZO-1), downregulating pro-inflammatory cytokines (IL-6, IL-8, TNF-*α*), and strengthening the intestinal barrier via enhancing epithelial defense and modulating the GM (Wang et al., 2018a). AAD triggered by GM dysbiosis post-antibiotic therapy, poses serious threat to human and animal health. However, *Lactobacillus plantarum* H-6 was found to modulate the colonic microbial composition in mice by increasing the abundance of *Lactobacillus* and *Akkermansia*, decreasing that of *Bacteroides*, downregulating the expression of pro-inflammatory factors (e.g., IL-1β, IL-6), and elevating the levels of L-tryptophan and LysoPC. These changes improve serum metabolism, thereby alleviating AAD (Yan et al., 2023). In addition, *Akkermansia muciniphila* was able to reduce the relative abundance of *Citrobacter* at the genus level, inhibit intestinal inflammation by up-regulating the expression of G protein-Coupled Receptor 109A (GPR109A) and Solute Carrier family 5 member 8 (SLC5A8) and down-regulating the expression of TNF-α, IFN-*γ*, IL-1β, and IL-6, and at the same time improve the down-regulation of ZO-1, Occludin, Claudin-4 (CLDN4), and Muc2 in AAD model mice, restore the intestinal barrier function and optimize intestinal health to prevent AAD (S. Liu et al., 2024). *Saccharomyces boulardii* can upregulate Serotonin Transporter (SERT) through activation of epidermal growth factor receptor and modulate GM to inhibit gut motility to alleviate IBS-D symptoms (Gu et al., 2022). The efficacy of other probiotics in modulating the GM for diarrhea treatment is summarized in Table 2, while the potential mechanisms underlying probiotic-mediated diarrhea management are illustrated in Figure 2.

**Table 2:** Basic studies on the potential mechanisms of probiotics against diarrhea.

Types of probiotics	Type of diarrhea	Effect on GM	Relief or treatment of symptoms	Reference
*Lactobacillus reuteri DSM* 17938	*E. coli*-induced diarrhea	Unclassified_Lachnospiraceae and *Anaerostipes* increased, *Escherichia_Shigella* decreased.	Restored the expression of inflammatory factors (IL-6, IL-10, TNF-α, and IFN-*γ*), reduced the colon inflammatory damage, maintained the integrity of the intestinal barrier, and regulated the composition of GM to alleviate diarrhea.	Wang D. et al. (2024)
*Lactobacillus paracasei*	Diarrhea caused by *E. coli* O8	Lactobacillus increased, Enterobacter decreased.	Elevated the TJ protein levels and downregulated proinflammatory cytokines IL-6, IL-1β, TNF-α, and p65, Myosin Regulatory Light Chain (MLC2), MLCK.	Ren et al. (2022)
*Lactobacillus plantarum* CCFM1143	Diarrhea caused by ETEC	*Odoribacter*, *Bifidobacterium*, *Allobaculum* and *Pediococcus* increased, *Blautia* and *Pseudomonas* decreased.	Reduced TNF-α, IFN-*γ* and IL-6 as well as jejunal damage, rebalanced the GM and modulated the production of SCFAs.	Yue et al. (2020)
*Lactobacillus reuteri* HCM2	Diarrhea caused by ETEC	*Lactobacillus* increased, Enterobacteriaceae decreased.	Inhibited the growth of ETEC, prevented ETEC infection-induced dysbiosis by stabilizing the relative abundance of the dominant bacteria.	Wang T. et al. (2018)
*Lactobacillus*	AAD	Muribaculaceae, *Bacteroides*, *Bifidobacterium*, *Lactobacillus* and *Akkermansia* increased, *Klebsiella*, *Parabacteroides*, and Clostridia_vadinBB60_group decreased.	Regulated the microbiota-SCFAs signaling cascade, improved SCFAs levels, inhibited the activation of the TLR4/NF-κB pathway, relieved the intestinal inflammation in AAD.	Xu et al. (2024)
*Limosilactobacillus fermentum* N-30	Diarrhea caused by rotavirus	Firmicutes increased, Bacteroidota and Proteobacteria decreased.	Improve diarrhea symptoms caused by rotavirus infection.	Murtaza et al. (2024)
*Bifidobacterium. bifidum* FSDJN7O5	Diarrhea caused by ETEC	*Bifidobacterium* and *Lactobacillus* increased, Escherichia–Shigella decreased.	Reduced the water content of the feces, restored the villi structure in the jejunum, and improved the content of SCFAs in the feces.	. Yang et al. (2021a)
*Bifidobacterium bifidum* G9-1	Phytohemagglutinin-induced diarrhea	Rikenellaceae decreased.	Inhibited the excessive proliferation of *E. coli*, restored the length of jejunum villi and relieved diarrhea symptoms.	Makizaki et al. (2019)
*Bifidobacterium animalis* subsp*. lactis* XLTG11	AAD	Muribaculaceae, *Bacteroides*, *Bifidobacterium*, *Lactobacillus* and *Akkermansia* increased, *Klebsiella*, *Parabacteroides* and Clostridia_vadinBB60_group decreased.	Increased the expression of TJ protein, inhibited the activation of TLR4/NF-κB signaling pathway, increased the level of anti-inflammatory cytokines, decreased the level of pro-inflammatory cytokines, increased the production of SCFAs, and decreased the permeability of the intestine.	Xu B. et al. (2022)
*Bacteroides uniformis* FGDLZ48B1 *and Bifidobacterium adolescentis* FHNFQ48M5	AAD	Restoring the diversity of GM.	Decreased IL-6 levels, restored occludin expression in the colon, increased Mucin-2 expression, and increased concentrations of acetic acid, propionic acid, isobutyric acid, and isovaleric acid in the cecum.	Guo et al. (2021)
*Weizmannia coagulans* WC10	AAD	*Bifidobacterium* and *Roseburia* increased.	Decreased diarrhea status score and fecal water content. Decreased the levels of serum enterotoxin and Diamine Oxidase (DAO), increased the expression of intestinal mucosal immune factors sIgA and occludin, decreased the expression of pro-inflammatory cytokines.	Wang Z. et al. (2024)
*Pediococcus pentosaceus* Li05	IBS-D	*Alloprevotella*, *Anaerotruncus* and *Mucispirillum* increased. [*Ruminococcus*] *gauvreauii*, *Dubosiella*, *Erysipelatoclostridium* and *Blautia* decreased.	It ameliorated intestinal and systemic inflammation by decreasing the levels of chemokines and pro-inflammatory cytokines. Regulated on Activation, Normal T cell Expressed and Secreted (RANTES), IL-1β, IL-7, and IL-18, and effectively reduced the expression of intestinal 5-Hydroy-tryptamine 3B (5-HT3B) receptor, regulated excessive intestinal motility and secretion in patients with IBS-D.	Wu et al. (2024)
*Lactiplantibacillus plantarum* ELF051	AAD	*Oscillospira* and *Prevotella* increased, *Allobaculum*, *Desulfovibrio* and *Akkermansia* decreasd.	Improved the pathological changes of colon tissue, down-regulated IL-1β and TNF-α, up-regulated IL-10, increased the level of intestinal SCFAs, and regulated TLR4/MyD88/NF-*κ*B and PI3K/AKT/NF-*κ*B signaling pathways, thereby reducing inflammation.	Liang et al. (2023)
*Lactiplantibacillus plantarum* 2-33	AAD	*Lactobacillus* increased, *Enterococcus* and *Bacillus* decreased.	Increased the levels of anti-inflammatory cytokines IL-4 and IL-10, reduced the levels of proinflammatory cytokines TNF-α and IFN-γ, and also adjusted carbohydrate metabolism, amino acid metabolism, restored energy metabolism to normal level, accelerated the recovery of intestinal bacterial structure in AAD mice, alleviated AAD.	Bao et al. (2022)

#### 3.1.2 Clinical experiments

Clinical studies have extensively documented the efficacy of probiotics in managing diarrhea. *Bifidobacterium bifidum* G9-1 was reported to reduce serum pro-inflammatory cytokine (Monocyte Chemoattractant Protein-1, IL-8 and Macrophage Inflammatory Protein-1β) levels and increase the abundance of *Bifidobacterium*, alleviating diarrhea in IBS-D patients (Tomita et al., 2023). Similarly, longum ES1 significantly lowered serum IL-6 and TNF-α levels in IBS-D patients compared to baseline (Caviglia et al., 2020). *Clostridium butyricum* ameliorated diarrhea in IBS-D patients by decreasing stool frequency and modulating GM composition (Sun et al., 2018). *Lactobacillus plantarum* LRCC5310 improved diarrhea and Vesikari scores in rotavirus-infected children while suppressing viral proliferation (Shin et al., 2020). *Alkalihalobacillus clausii 088AE* exerts a therapeutic effect on diarrhea in children, adolescents, and adults. Specifically, in the treatment of AAD, it has been demonstrated to be safe and effective in reducing diarrhea episodes and alleviating associated severe symptoms, such as abdominal discomfort, pain, bloating, and flatulence (Maity and Gupta, 2021). However, supplementation with *Bifidobacterium breve* BB05 can partially restore the disrupted GM at both the phylum and genus levels, notably by elevating the abundances of *Bifidobacterium* and *Roseburia*. In the probiotic-supplemented group, fecal 5-HT concentration was increased, whereas levels of acetylcholine, epinephrine, and norepinephrine were reduced—suggesting that *Bifidobacterium breve* BB05 may alleviate anxiety and diarrhea by BGMA (Wang Y. et al., 2024).

**Figure 3:**
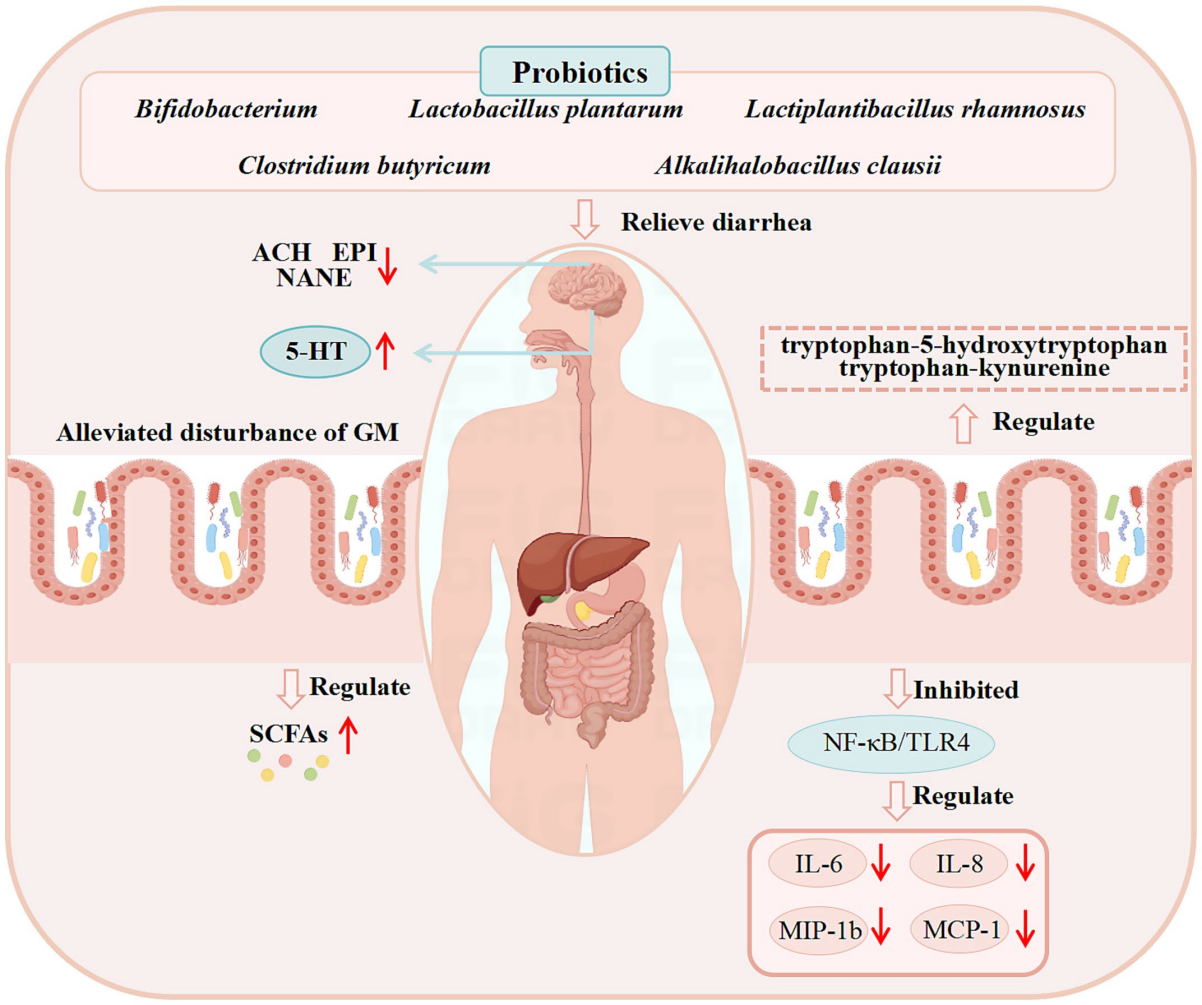
Demonstrated mechanisms of probiotics against diarrhea from clinical studies. (These potential mechanisms mainly involve GM, SCFAs, the immune system, and BGMA).

Most existing literature has only summarized the therapeutic effects of probiotics on clinical diarrhea (focusing on diarrhea alleviation) and their safety profiles in patients, with relatively few studies investigating their specific efficacy and underlying mechanisms of action. Supplementary details regarding the clinical application of probiotics for diarrhea treatment and their corresponding mechanisms are provided in Table 3, while the potential mechanisms through which probiotics may exert anti-diarrheal effects in clinical settings are illustrated in Figure 3.

**Table 3:** Clinical studies on the potential mechanisms of probiotics against diarrhea.

Types of Probiotics	Type of diarrhea	Effect on GM	Relief or treatment of symptoms	Reference
Combined *B. infantis, L. acidophilus, E. faecalis,* and *B. cereus* tablets	Chemotherapy-induced diarrhea in patients with rectal cancer	*Streptococcus*, *Blautia* and *Bifidobacterium* increased.	Alleviated chemotherapy-induced diarrhea in Colorectal Cancer (CRC) patients by modulating the GM and promoting SCFA production.	Huang et al. (2023)
*Lactobacillus plantarum* CCFM1143	Chronic Diarrhea	*Akkermansia*, *Anaerostipes*, *Terrisporobacter*, *Escherichia*, and *Shigella* increased, *Bacteroides*, *Eggerthella*, *Lachnoclostridium*, and *Lachnospira* decreased.	Reduced the clinical symptoms of chronic diarrhea. In addition, it inhibited the elevation of IL-6 and the reduction of motilin; and regulated the production of SCFAs.	Yang et al. (2021b)
*Lacticaseibacillus paracasei* Zhang, *Lactiplantibacillus plantarum* p-8, and *Bifidobacterium animalis* subsp. *lactis* V9	Chronic diarrhea	*Dysosmobacter welbionis* and *Faecalibacterium prausnitzii* increased, *Megamonas funiformis* decreased.	Alleviated diarrhea by modulating the tryptophan-5-hydroxytryptophan and tryptophan-kynurenine pathways, and improved the patients’ Bristol Stool Scale scores, frequency of defecation, and urgency to defecate.	Guo et al. (2024)
*Lactiplantibacillus plantarum* CJLP243	Functional diarrhea	*Leuconostoc* increased.	Improved diarrhea symptoms.	Jung M. et al. (2022)
*Lacticaseibacillus rhamnosus* LRa05	AAD	*Faecalibacterium*, *Lachnospira*, *Parabacteroides*, *Phascolarctobacterium*, *Fusicatenibacter*, *Alistipes*, *Coprococcus*, *Oscillibacter*, *Parasutterella*, and *Megamonas* increased.	Relieved adverse symptoms, regulated the inflammatory response.	Niu et al. (2024)
*Lactiplantibacillus plantarum* APsulloc 331261	IBS-D	Firmicutes increased, Bacteroidetes decreased.	Reduced the severity and frequency of abdominal pain, bloating, and feeling of incomplete evacuation.	Jung K. et al. (2022)
*Bifidobacterium animalis* subsp. *lactis* BLa80	Acute diarrhea in children	*Bifidobacterium breve* and *Lactobacillus murinus* increased, *Bifidobacterium longum* decreased.	Reduced duration of diarrhea, accelerated improvement in stool consistency and alteration of the gut microbiome.	Chen K. et al. (2024)

### 3.2 FMT

FMT is a procedure that involves transferring GM from healthy donors into the gastrointestinal tract of patients to restore a balanced microbial community and treat diseases, particularly those associated with gut dysbiosis such as diarrhea (Almeida et al., 2022; Li et al., 2022).

The therapeutic efficacy of FMT in diarrhea alleviation is closely linked to the modulation of GM composition. On one hand, FMT reintroduces a healthy microbial community that competes for ecological niches in the gastrointestinal tract, thereby suppressing pathogen colonization-this process further facilitates the restoration of immune function and mitigates host tissue damage. On the other hand, FMT aids in replenishing essential metabolites for host metabolism, such as SCFAs, antimicrobial peptides, bacteriocins, and BAs (Ademe, 2020). IBS-D is a common gastrointestinal disorder and is characterized by altered GM, especially involving Firmicutes and Bacteroidetes (Mei et al., 2021; Zhen et al., 2021). However, FMT can reduce intestinal permeability and alleviate the diarrheal effects of IBS-D by modulating GM disorders and affecting GM-produced metabolites such as increasing the production of SCFAs (Lin et al., 2021; Singh et al., 2022; Song et al., 2023). *Clostridium difficile* infection (CDI) is considered a common cause of AAD (Bosnjak et al., 2023; Tubau-Juni et al., 2023). One study demonstrated that FMT administration to children with recurrent CDI enhanced GM diversity while driving shifts in GM composition and function toward those of the donor (Fareed et al., 2018). During the weaning transition, piglets are prone to diarrhea, which is related to the damaged state of the microbiome and immature immune system (Han et al., 2024). In diarrheal piglets infected with *E. coli* K88, the application of FMT increased the number of beneficial bacteria in the gut and reduced the number of harmful bacteria, and further research found that FMT triggered intestinal mucosal autophagy and reduced the damage of *E. coli* K88 to the intestinal barrier (Cheng et al., 2018).

Currently, research on the underlying mechanisms of FMT remains limited. Available evidence suggests that alterations in the GM following FMT play a significant role in the pathogenesis of diarrhea. However, several studies indicate that the therapeutic efficacy of FMT may be constrained. Additionally, to date, FMT has been predominantly investigated for the treatment of CDI and diarrhea-predominant IBS-D, while its efficacy in diarrhea of other etiologies remains less established. Thus, further clinical trials are warranted to validate the potential benefits of FMT across diverse forms of diarrhea and to better define its role in managing diarrhea-related disorders. It is also important to note that the limited efficacy of FMT in some cases of diarrhea may be attributable to insufficient donor-recipient matching, among other factors, highlighting the need for more personalized approaches in future studies.

#### 3.2.1 Basic experiment

Diarrhea in animals, triggered by multiple etiologies, is highly prevalent and remains a major challenge afflicting the animal husbandry industry. To address this issue, FMT-an emerging therapeutic technology-has been increasingly applied to the treatment of animal diarrhea in recent years.

Advances in modern genetics and enhanced sow reproductive performance have facilitated the widespread implementation of artificial lactation systems in commercial swine production. However, these systems are linked to a high incidence of diarrhea in piglets. To tackle this challenge, researchers have utilized FMT as a therapeutic strategy to alleviate diarrhea induced by artificial feeding. Results demonstrated that FMT modulates the composition of colonic microbiota and its metabolites, promotes tryptophan metabolism and 5-hydroxyindoleacetic acid (5-HIAA) production, enhances intestinal mucosal barrier function, inhibits the activation of the Jun N-terminal kinase (JNK) pathway and the expression of matrix metalloproteinases (MMPs), reduces the secretion of proinflammatory cytokines and chemokines, and ultimately alleviates artificial feeding-induced diarrhea in piglets (Han, 2023). Bovine viral diarrhea virus (BVDV) is widespread throughout the world and has caused significant economic losses to animal husbandry (Pang et al., 2023). BVDV infection significantly decreased the diversity and changed the composition of GM in mice. However, after FMT, BVDV RNA and protein levels in duodenum, jejunum, spleen and liver were significantly inhibited, Interferon-*α* (IFN-α) and Interferon-*β* (IFN-β) mRNA levels were increased, and Interferon Regulatory Factor 1 (IRF1) and Interferon Regulatory Factor 7 (IRF7) mRNA levels were increased. The expression of Toll-Like Receptor 7 (TLR7) and Toll-Like Receptor 9 (TLR9) was restored, the proportion of Cluster of Differentiation 3 (CD3) and CD8 T cells was restored, the expression of ZO-1 protein was increased, and the proliferation of Peripheral Blood Leukocytes (PBL) was restored (Zhang Z. et al., 2024). FMT significantly alleviates symptoms of IBS-D, potentially through modulating the 5-HT signaling pathway within the BGMA. It was found that after FMT, the mental condition of IBS-D mice was improved, the diarrhea was improved, and the fecal water content was significantly reduced. Additionally, the expression levels of 5-HT and SP in brain tissue and serum were significantly decreased, the expression levels of SERT and 5-Hydroxytyryptamine Receptor 4 (5-HT4R) proteins in colon and brain tissues were increased, and the expression levels of Tryptophan Hydroxylase 1 (THP1) and (5-Hydroxytyryptamine Receptor 3) 5-HT3R proteins were significantly reduced (Ouyang et al., 2022).

**Figure 4:**
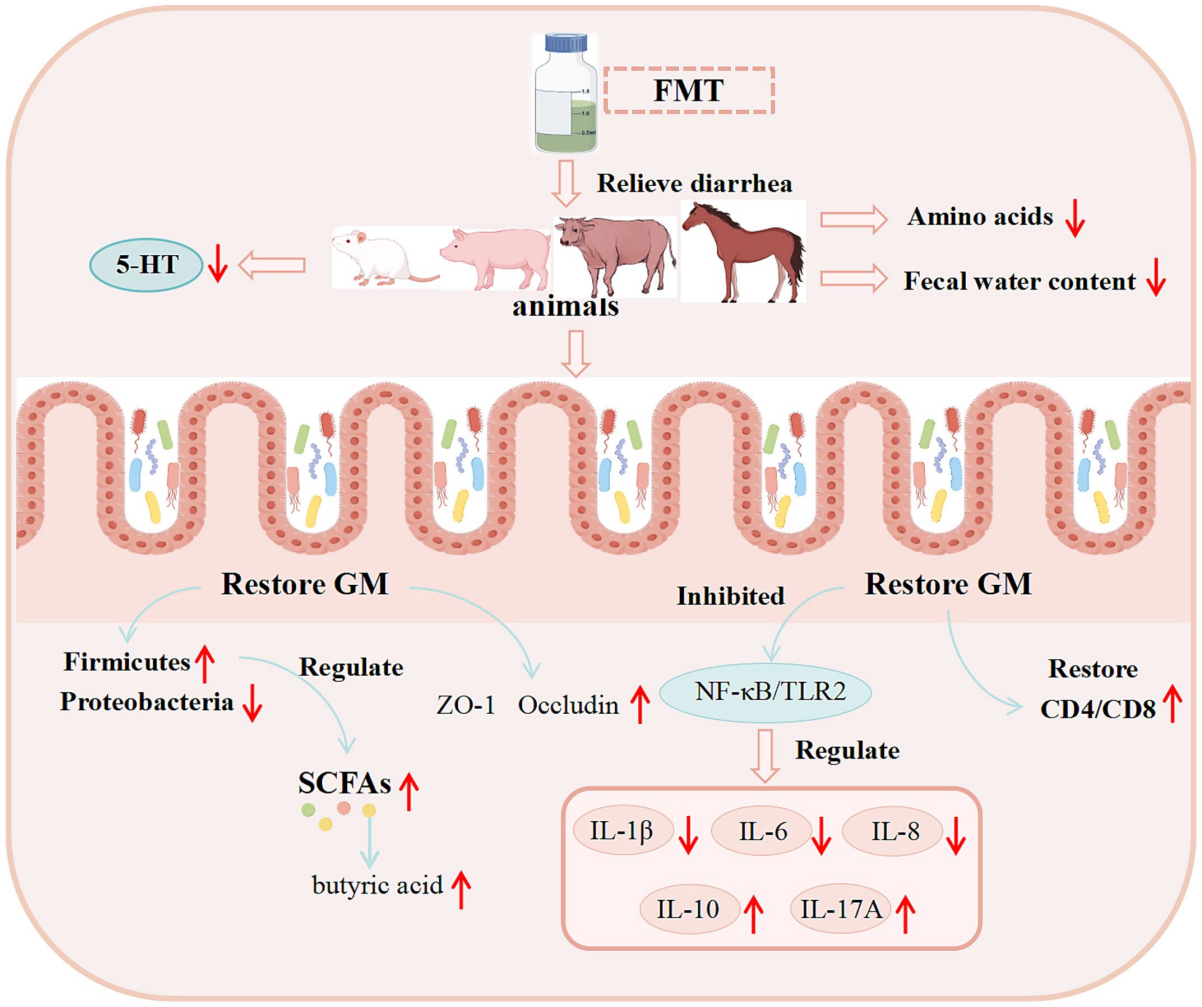
Potential mechanisms of FMT in treating diarrhea revealed by basic research. (These potential mechanisms mainly involve GM, SCFAs, the immune system, and intestinal barrier function).

Basic experimental data on the use of FMT for diarrhea treatment in other studies are presented in Table 4, while the potential mechanisms underlying FMT’s therapeutic effects on diarrhea are illustrated in Figure 4.

**Table 4:** Basic studies on the application of FMT technique in the treatment of diarrhea.

Type of diarrhea	Donor	Changes in microbiota	Relief or treatment of symptoms	Reference
Horse with diarrhea	Healthyage-matched control horses	Verrucomicrobia increased and Proteobacteria decreased.	Reduced the severity of diarrhea.	McKinney et al. (2020)
Foals with diarrhea	Healthy calves	Verrucomicrobiota and *Akkermansia* increased.	Alleviated the symptoms of weaning diarrhea in calves.	Bell et al. (2024)
Cynomolgus monkeys with diarrhea	Healthy human donors	Firmicutes and *Lactobacillus* increased, *Lactobacillus fermentum* and *Lactobacillus ruminis* CAG_367 increased.	Increased serum levels of IL-10 and decreased levels of IL-6, IL-8, IL-1β and IFN-γ in monkeys with chronic diarrhea.	Tian et al. (2022)
Calves suffering from intractable diarrhea	Healthy calves	*Lactobacillus*, Veillonellaceae, *Selenomonas*, *Acidaminococcus*, and *Collinsella* increased.	Increased the content of SCFAs (especially butyric acid) and medium-chain fatty acids (e.g., octanoic acid) after FMT treatment. Decreased the fecal content of most amino acids in successful recipients.	Islam et al. (2022)
Post-weaning diarrhea in piglets	Healthy Tibetan pigs	Firmicutes, Euryarchaeota, and Tenericutes increased, Proteobacteria and Melainabacteria decreased. *Lactobacillus* and *Methanobrevibacter* increased, *Campylobacter* decreased.	Reduced the incidence of diarrhea, which attenuated the reduction of CD4 T cells and CD4/CD8 ratio in peripheral blood. Down-regulation of mRNA expression of Toll-Like Receptor 2 (TLR2) and NF-*κ*B.	Tang et al. (2020)
IBS-D rats	Healthy rat	Firmicutes and *Bacteroides* increased, Proteobacteria and *Prevotella* decreased.	Inhibited visceral hypersensitivity and regulated GM balance to relieve diarrhea.	Jiang C. Y. et al. (2024)
Oligofructose-induced diarrhea in horses	Healthy horse	Patescibacteria and Planctomycetota increased, Proteobacteria, Desulfobacterota, and Fusobacteriota decreased. *Streptococcus* and *Lactobacillus* decreased.	Decreased body temperature and diarrhea score, and increased fecal pH, decreased inflammatory responses such as increased serum Lipopolysaccharide (LPS), IL-17A, lactic acid and total protein.	Tuniyazi et al. (2024)

#### 3.2.2 Clinical experiments

FMT has emerged as a novel clinical strategy for treating diarrhea. As an intestinal microecological therapy with proven efficacy, FMT entails the transfer of GM from healthy donors to patients with diarrhea, which modulates GM composition, restores the intestinal mucosal immune barrier, and thereby exerts therapeutic effects.

CDI is the main cause of nosocomial infectious diarrhea and a high proportion of clinical cure rates have been achieved by restoring the GM with FMT in CDI therapy (Chen and Chiu, 2022; Roshan et al., 2020). Shao et al. found a significant increase in a diversity of GM in CDI patients after FMT, with GM composition more similar to that of healthy donors, increased the abundances of families Ruminococcaceae, Prevotellaceae, Coriobacteriaceae, Porphyromonadaceae, Bacteroidaceae, Bifidobacteriaceae, and Eubacteriaceae, and reduced the abundance of Enterobacteriaceae, Veillonellaceae, Enterococcaceae, and Peptostreptococcaceae (Wei et al., 2022). Clinically, FMT alleviates IBS-D symptoms and improves patients’ quality of life by restoring a balanced GM (Fu and Huang, 2022). Studies have shown that GM and SCFAs in patients with IBS-D differ from those of donors at baseline, such as decreased levels of Actinobacteria and *Bifidobacterium* and increased levels of Bacteroidetes and Proteobacteria, however these differences gradually return to normal after 3 weeks after FMT, while patients also have improved symptoms and quality of life of IBS-D during the same period (Mazzawi et al., 2019). However, some studies have also shown conflicting results. For example, studies in the treatment of IBS-D have shown that both FMT and placebo recipients showing improvements in irritable bowel syndrome-Severity Scoring System (IBS-SSS) and irritable bowel syndrome-Quality of Life (IBS-QOL) scores and reporting improvements in fecal morphology, however, no differences were found between the two groups (Aroniadis et al., 2019). Therefore, more research is needed to determine the efficacy of FMT for IBS-D.

**Figure 5:**
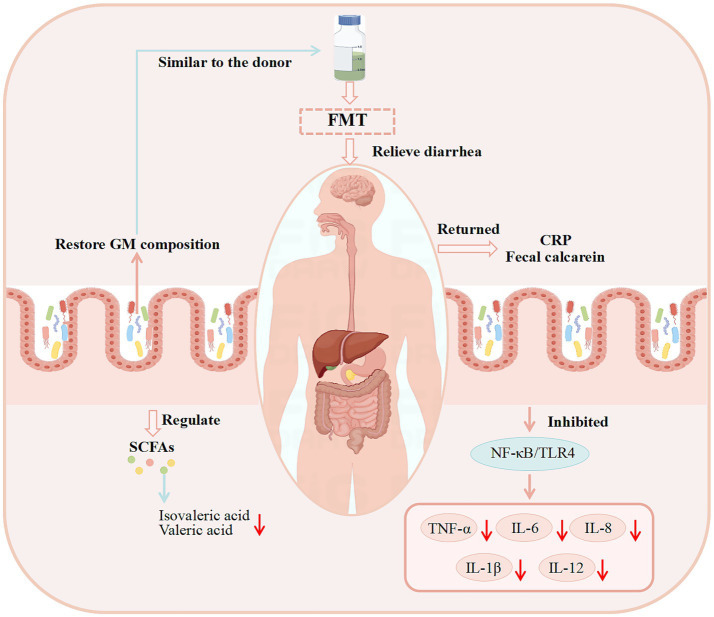
Observed outcomes and proposed mechanisms of FMT for diarrhea in clinical studies. (These potential mechanisms mainly involve GM, SCFAs, and immune system).

Currently, FMT has shown expanding clinical applications across various diseases. However, clinical evidence supporting FMT for diarrhea remains limited, with existing studies reporting inconsistent therapeutic outcomes. Most available literature has documented improvements in clinical symptoms, FMT safety, and the efficacy and duration of single or multiple transplantation regimens in diarrhea patients, while studies investigating its specific mechanisms of action remain scarce. Additional information on FMT-induced symptomatic improvements and mechanisms in diarrhea treatment is presented in Table 5, and the potential mechanisms underlying FMT’s clinical efficacy in diarrhea are illustrated in Figure 5.

**Table 5:** Clinical study on the application of FMT technique in the treatment of diarrhea.

Type of diarrhea	Donor	Changes in microbiota	Relief or treatment of symptoms	Reference
IBS-D	Healthy 36-year-old men	*Faecalibacterium*, *Eubacterium* and *Escherichia* decreased.	Relieved diarrhea and anxiety symptoms in IBS-D patients and reduced fecal isovaleric and valeric acid levels.	Lin et al. (2021)
*Clostridium difficile* infection (CDI)	Healthy donor	Lactobacillaceae, Ruminococcaceae, Desulfovibrionaceae, Sutterellaceae and Porphyromonadaceae increased, Enterobacteriaceae and Veillonellaceae decreased.	Decreased serum proinflammatory cytokines (TNF-α, IL-1β, IL-6, IL-8 and IL-12), returned CRP and fecal calcarein to normal. Increased LL-37 in plasma of successfully treated patients were monitored 3 months after FMT.	Konturek et al. (2016)
AAD	Healthy donor	Firmicutes, Bacteroidota, and Actinobacteriota increased, Proteobacteria decreased. *Bacteroides* and *Faecalibacterium* increased, *Escherichia*-*Shigella* and *Veillonella* decreased.	Reduced inflammatory markers IL-8 and CRP and alleviated diarrhea symptoms in patients.	Wang L. et al. (2024)
IBS-D	FMT was prepared from one or two healthy unrelated donors	*Lawsonibacter* increased, *Ruminococcus gnavus* decreased.	Relieved the patients’ abdominal distension and general symptoms.	Yau et al. (2023)
IBS-D	Screening of eligible faecal donors, and matched by donor-recipient	*Gemella* in donor-recipient-matched group and *Acidovorax* and *Klebsiella* in random-donor group were decreased.	Compared with random-donor FMT, donor-recipient-matched FMT significantly improves the clinical symptoms of patients with IBS-D.	Zhang Y, et al. (2024)
IBS-D	Healthy donor	*Weissella*, *Bacteroides*, *Escherichia*-*Shigella*, *Akkermansia*, *Enterococcus*, *Parabacteroides*, *Collinsella* and *Dorea* increased, *Streptococcus*, *Lactobacillus*, *Romboutsia*, *Bifidobacterium*, *Subdoligranulum*, *Pediococcus*, *Blautia*, *Faecalibacterium*, and *Fusobacterium* decreased.	Improved the patient’s QOL, and also improved Hamilton anxiety scale and Hamilton depression scale scores, and was effective in the following 4 dimensions: interference with activities, health concerns, food avoidance, and interpersonal relationships.	Huang et al. (2022)

### 3.3 Bacteriophage therapy

Bacteriophages, viruses that specifically infect and lyse bacteria, offer a promising therapeutic strategy by precisely targeting pathogenic bacteria while preserving the commensal GM (Chen H. et al., 2023; Chen X. et al., 2023; Chen J. et al., 2023; Strathdee et al., 2023).

Antibiotics have always been the cornerstone of treating diarrhea, but rising antimicrobial resistance (AMR) has diminished their efficacy (Baran et al., 2023). Moreover, antibiotics disrupt the commensal GM, leading to dysbiosis and increased susceptibility to recurrent infections (Ramirez et al., 2020). Phage therapy, which uses viruses to specifically infect and kill bacteria, has re-emerged as a promising alternative due to its specificity, self-replicating nature, and ability to disrupt biofilms (Duan et al., 2022).

Multiple *in vitro* studies have demonstrated the effectiveness of phage therapy for treating diarrheal pathogens. Research has shown that a phage cocktail targeting *E. coli*, such as a combination of six bacteriophages, can reduce bacterial load by 3 log CFU/mL in vitro and effectively inhibit biofilm formation (Youssef et al., 2025). In addition, a 2023 study utilized a resource library termed the gut phage isolate collection (GPIC)—composed of bacteriophages isolated from healthy human guts—to demonstrate that a bacteriophage cocktail targeting *Bacteroides fragilis* significantly reduced the abundance of the target bacteria in in vitro fecal cultures, highlighting the potential of bacteriophages in modulating the GM (Shen et al., 2023). In animal model studies, phage therapy has also shown promising effects. Research has shown that the microencapsulated bacteriophage A221 is as effective as the antibiotic florfenicol in treating piglet diarrhea models (Youssef et al., 2025). In addition, phage cocktail therapy targeting *Klebsiella pneumoniae* associated with inflammatory bowel disease can alleviate intestinal inflammation and tissue damage in mouse models (Fuerte-Stone and Mimee, 2022). These studies indicate that bacteriophages can not only effectively reduce the load of pathogenic bacteria, but also alleviate the inflammatory response and tissue damage caused by it.

In recent years, clinical trials of phage therapy for diarrhea have also made some progress. A phase 1 clinical trial in 2022 tested two bacteriophages targeting *Klebsiella pneumoniae* associated with inflammatory bowel disease on 18 healthy volunteers. The results showed that when taken together with antacids such as CaCO₃, the bacteriophages not only survived at high levels but also remained active throughout the gastrointestinal tract without affecting the resident GM. All participants did not experience any serious treatment-related adverse events, laying the foundation for further research in patients (Federici et al., 2022).

Although these studies indicate that bacteriophages have great potential in treating diarrhea, a critical translational challenge involves phage instability in the harsh gastrointestinal environment. Gastric acidity and digestive enzymes rapidly denature phage particles, compromising therapeutic efficacy. Advanced encapsulation strategies using electrospun fibers, liposomes, or pH-responsive hydrogels are being developed to shield phages during transit and ensure targeted colonic release (Yang Y. et al., 2023; Yang Q. et al., 2023). Additionally, the field must address the complexity of phage ecology, particularly the potential for temperate phages to facilitate horizontal gene transfer of virulence or resistance genes. Careful selection of obligately lytic phages is therefore essential for clinical safety and efficacy (Gummalla et al., 2023).

#### 3.3.1 Basic experiment

Diarrhea is now a significant public health concern. Consequently, bacteriophage therapy has emerged as a promising therapeutic strategy. Phages modulate the composition and abundance of the GM, which in turn alters the expression of intestinal proteins and inflammatory factors, ultimately alleviating various forms of diarrhea.

The mechanism of action of bacteriophages against diarrhea is multifaceted. It begins with the specific lysis of bacterial pathogens, which in turn drives the recovery of healthy GM. This rebalancing directly leads to a reduction in inflammation, an enhancement of the intestinal barrier, and a positive regulation of the immune response, collectively alleviating the symptoms and pathology of diarrhea. Specifically, bacteriophage vB_Ecos_ULIM2 effectively lysed F18 ETEC strain (Navez et al., 2023), ZC22 bacteriophage specifically targeted and reduced the load of *Salmonella typhimurium* in organs (Sun et al., 2025), and broad-spectrum cocktail reduced fecal *E. coli* count by 1.33 logarithmic units (Sun et al., 2025).

In addition to direct killing, bacteriophages also significantly regulate the GM to restore health: (1) Reduce pathogenic bacteria: Multiple studies have shown that bacteriophages effectively reduce the abundance of pathogenic families such as Enterobacteriaceae (Li et al., 2024; Mao et al., 2023) and specific genera such as Shigella, Clostridium, and Desulfovibrio (Chen et al., 2025; Choi et al., 2023). (2) Promotion of beneficial bacteria: A key finding is that targeted phage therapy can reduce or even promote the growth of beneficial bacteria. Research consistently reports an increase in the abundance of *Lactobacillus* (Canibe et al., 2022; Castro et al., 2022; Chen J. et al., 2025; Choi et al., 2023; Feng et al., 2025; Mao et al., 2023) and *Bifidobacterium* (Chen J. et al., 2025; Choi et al., 2023), which is crucial for gut health. (3) Increasing diversity: Phage cocktails can increase the richness and diversity of microorganisms that are infected and destroyed (such as Chao1 index) (Zeng et al., 2021).

Continuous treatment with bacteriophages can also lead to significant reductions in pro-inflammatory cytokines such as IL-1β, IL-6, IL-8, and TNF-*α* (J. Chen et al., 2025; Choi et al., 2023; Dong et al., 2024; Kim et al., 2022; Sun et al., 2025; Zeng et al., 2021). On the contrary, they can increase the levels of anti-inflammatory cytokines such as IL-10 (Zeng et al., 2021). A reduction in inflammation often correlates with decreased intestinal damage. Bacteriophages contribute to the restoration of intestinal barrier integrity, they upregulated the expression of TJ proteins, including ZO-1, Occludin, and Claudin-1/3 (Choi et al., 2023; Dong et al., 2024; Feng et al., 2025; Kim et al., 2022). This will lead to a decrease in intestinal permeability (Kim et al., 2022). In addition, there will also be improvements in intestinal morphology, with studies showing an increase in villus height and a decrease in crypt depth (Chen J. et al., 2025; Choi et al., 2023; Zeng et al., 2021), indicating enhanced nutrient absorption and intestinal health.

In addition to the above, bacteriophages can regulate the host’s immune response, including enhancing specific immunity (increasing IgA and IgG levels) and non-specific immunity (such as increasing IFN-*γ* and lysozyme activity) (Alomari et al., 2021). Bacteriophages can also indirectly affect the intestinal environment, and some therapies lead to an increase in SCFAs (Dong et al., 2024), which are beneficial metabolites produced by intestinal bacteria, supporting barrier function and reducing inflammation.

**Figure 6:**
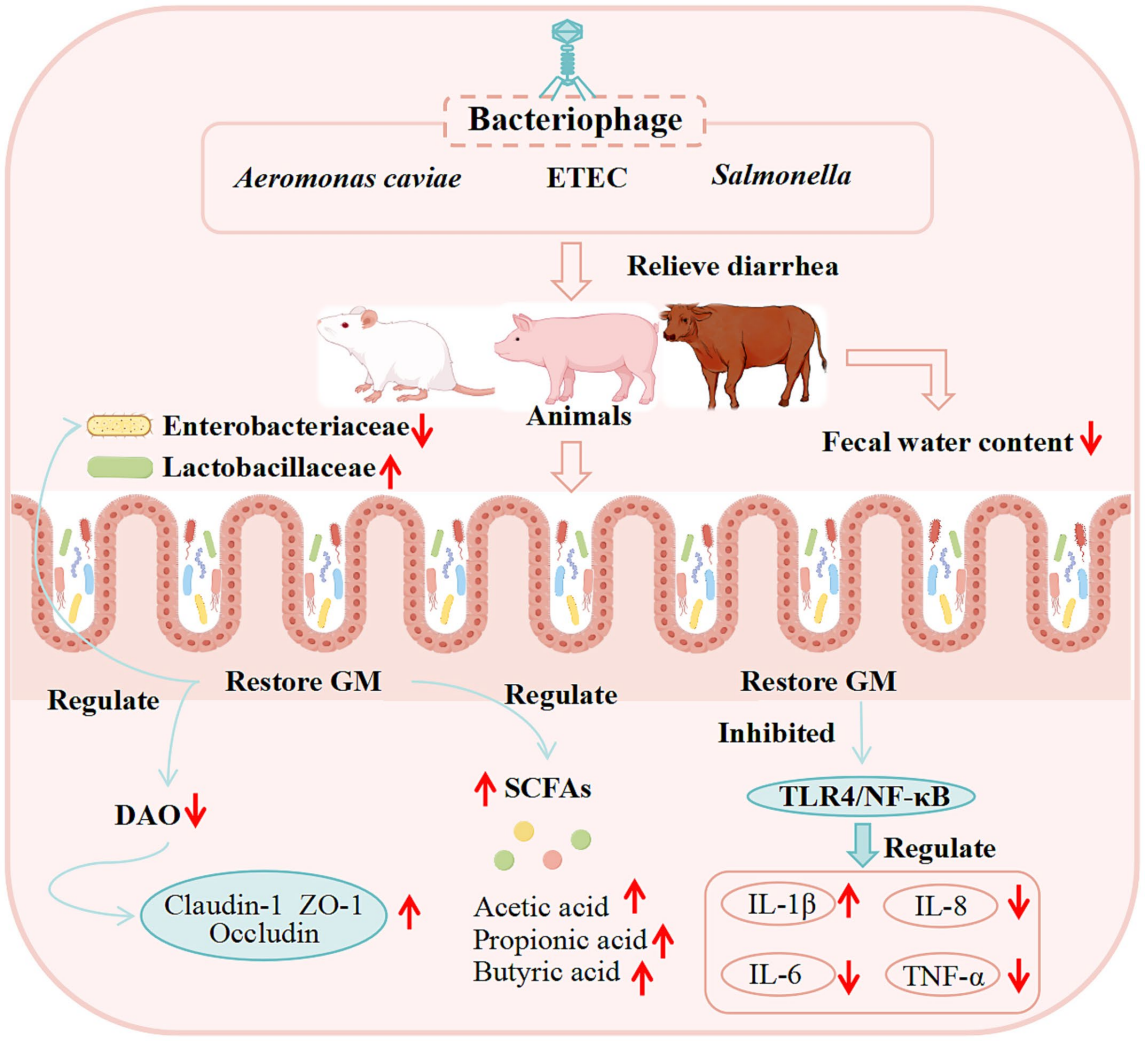
Potential mechanisms of bacteriophage therapy for diarrhea from basic studies. (These potential mechanisms mainly involve GM, SCFAs, the immune system, and intestinal barrier function).

The specific basic experiments of bacteriophages in the treatment of diarrhea were shown in Table 6, and the potential mechanism of bacteriophages in the treatment of diarrhea were shown in Figure 6.

**Table 6:** Basic studies on the potential mechanisms of bacteriophages against diarrhea.

Types of bacteriophages	Type of diarrhea	Effect on GM	Relief or treatment of symptoms	Reference
Microencapsulated Phage A221	*E. coli*-induced PWD (Post-Weaning Diarrhea)	Reduced Enterobacteriaceae abundance in duodenum to 0.64%; Increased Lactobacillaceae and Oscillospiraceae.	Reduced bacterial load in jejunal lymph nodes, cecum, and spleen, and alleviated intestinal lesions (villi atrophy, gland degeneration, bleeding).	Mao et al. (2023)
Phage ZK22	*Salmonella*-induced diarrhea	Highly specific to *Salmonella Typhimurium*; no significant disruption to normal GM expected due to narrow host range.	Increased survival rate in mice; reduced bacterial load in blood, heart, liver, and spleen; alleviated inflammatory response (IL-2, IL-6, TNF-α).	Sun et al. (2025)
Unclassified *Caudoviricetes*, *Siphoviridae*	Early-onset diarrhea	Increased viral heterogeneity; reduced *Clostridium perfringens* and *Escherichia*; constrained bacterial composition.	Reduced NEC severity, intestinal inflammation, and levels of IL-1β and IL-8; decreased abundance of pathobionts.	Spiegelhauer et al. (2025)
Mixed bacteriophage (Targeting *Salmonella*, *E. coli*, *Clostridium perfringens*, *S. aureus*)	Post-weaning diarrhea	Altered relative abundance of Firmicutes, Bacteroidetes, and Tenericutes.	Reduced feed/gain ratio and diarrhea incidence; Enhanced intestinal morphology (increased villus height, decreased crypt depth); modulated inflammatory response (decreased IL-1*β*, TNF-α; increased IL-10); enhanced intestinal barrier function (increased ZO-1, Claudin-1, Occludin).	Zeng et al. (2021)
φ26, φ27, φ29 (All belonging to Myoviridae)	*E. coli*-induced diarrhea	Reduced pathogenic *E. coli*; no effect on commensal *E. coli*	Reduced duration of diarrhea; enhanced specific (IgA, IgG) and nonspecific (IFN-*γ*, lysozyme) immune response; reduced inflammatory damage; maintained intestinal barrier integrity.	Alomari et al. (2021)
Myovirus phage vB_AccP_PAc	*Aeromonas caviae*-induced diarrhea	Lactobacillaceae increased	Alleviated diarrhea, reduced inflammatory cytokines, increased TJ molecules, and improved intestinal barrier function.	Feng et al. (2025)
Bacteriophage EK99P-1	ETEC K99-induced diarrhea	Reduction of ETEC K99 colonization	Restored intestinal barrier integrity (ZO-1, occludin, claudin-3), reduced intestinal permeability, decreased pro-inflammatory cytokines (IL-8, MCP-1, IL-1*β*).	Kim et al. (2022)
Broad-spectrum phage cocktail (vs. multiple pathogens)	Non-sanitary environment diarrhea	Decreased Proteobacteria, *Desulfovibrio*, *Escherichia-Shigella*, *Clostridium* spp.; increased *Eubacterium* and *Lactobacillus* spp.	Increased ADG, G/F; decreased fecal score; decreased serum IL-1β, IL-6, TNF-α; increased intestinal barrier function; improved microbiota diversity.	Chen J. et al. (2025)
Microencapsulated phage cocktail (NJ12 + EP01)	Mixed *E. coli* O157: H7 and *Salmonella Typhimurium*-induced diarrhea	Reduced the relative abundance of *Enterobacteriaceae*	Reduced diarrhea incidence and severity; increased fecal score; decreased bacterial load in jejunum; attenuated intestinal inflammation and damage; improved weight gain in weaned piglets.	Li et al. (2024)
Bacteriophage cocktail (targeting *E. coli*, *Salmonella*, *Clostridium perfringens*)	Post-weaning diarrhea (mainly *E. coli* and *Clostridium* spp. induced)	Decreased Proteobacteria; *Escherichia*-*Shigella*; increased *Eubacterium*; *Lactobacillus* spp.; *Bifidobacterium* spp.; decreased *Clostridium* spp.; coliforms; *Desulfovibrio*.	Improved growth performance (final BW, ADG, G/F); reduced pro-inflammatory cytokines (IL-1β, IL-6, TNF-α); decreased myeloperoxidase (MPO) and zonulin; enhanced antioxidant capacity (increased SOD, decreased MDA); improved intestinal morphology, reduced diarrhea incidence and fecal score.	Choi et al. (2023)
Unclassified *Caudoviricetes*, *Siphoviridae*	Early-onset diarrhea	Reduced *Clostridium perfringens* and *Escherichia*	Reduced NEC severity, intestinal inflammation, and levels of IL-1*β* and IL-8; decreased abundance of pathobionts.	Kreis and Soutourina (2022)
Bacteriophage cocktail (*Salmonella*, *E. coli*, etc.)	ETEC-induced diarrhea	Increased *Lactobacillus* concentration	Improved average daily gain, feed intake, and nutrient digestibility; enhanced villus height in duodenum and jejunum.	Castro et al. (2022)
Bacteriophage cocktail (*E. coli* strains K88, K99, F18, F41, 987P, O78)	ETEC-induced diarrhea	Increased the relative abundance of Bacteroidota and Muribaculaceae, decreased the relative abundance of Verrucomicrobiota and Akkermansiaceae	Reduced serum DAO level and increased the expression of Claudin-1, Occludin, and ZO-1. Decreased TNF-α, IL-1β and IL-6 levels, and inhibited TLR-4/NF-κB pathway activation induced by ETEC infection. Moreover, the bacteriophage administration increased the levels of acetic acid, propionic acid, butyric acid, and total SCFAs.	Dong et al. (2024)

#### 3.3.2 Clinical experiments

At present, in addition to probiotics, FMT and other important treatments for diarrhea, phage therapy is also used in clinical diarrhea diseases.

Based on a randomized, double-blind, placebo-controlled clinical trial conducted in Bangladesh, oral bacteriophage therapy was evaluated as a method for treating acute bacterial diarrhea in children. These studies aim to evaluate the safety, *in vivo* kinetics, and clinical efficacy of a customized T4 like *E. coli* phage cocktail and a commercial Russian phage product (Microgen ColiProteus). A total of 120 male children aged 6–24 months hospitalized for acute diarrhea received phage or placebo treatment, as well as standard oral rehydration and zinc therapy. The results indicate that oral phage administration is safe, with no evidence of systemic phage exposure, endotoxin release, or immune response (such as anti phage or anti LPS antibodies) detected, and no serious adverse events or systemic inflammatory reactions observed. These studies emphasize the inherent instability of the gut microbiota in Bangladeshi children, which should be considered in future research on the association of microbiota diseases (Sarker et al., 2017; Sarker and Brüssow, 2016; Sarker et al., 2016). Another study conducted safety testing of phage therapy, which was designed as a single center, randomized, placebo-controlled study. Fifteen healthy volunteers received higher doses of bacteriophages (dose A, 105 PFU/ml), lower doses of bacteriophages (dose B, 103 PFU/ml), and placebo (dose C). The subjects were randomly assigned to one of the following treatment sequences: ABC, BCA, or CAB. During the study, participants provided all fecal samples produced daily. The incidence of adverse events in the high-dose phage group was comparable to that in the low-dose and placebo groups. Ultimately, no adverse events were found to be related to phage administration (Bruttin and Brüssow, 2005).

Although phage therapy is safe and has the potential to serve as an alternative to antibiotic treatment for drug-resistant infections, its efficacy in treating diarrhea has not been confirmed in controlled trials. In the future, we need to conduct pre-screening of phage susceptibility and pathogen dominance, and further fundamental research on phage bacterial dynamics in the human gut. The reason for poor efficacy in the diarrhea test described above may be that some patients have low abundance of the target pathogen (*E. coli*). There are other pathogens that bacteriophages do not target, such as streptococcus. Possible issues with phage stability, dosage, or delivery to the site of infection. Future research on phage therapy for diarrhea should incorporate more rigorous randomized controlled trials, improved phage characterization, comprehensive sensitivity testing, and optimized dosage regimens.

## 4 Discussion

Diarrhea, induced by diverse pathogens or contributing factors, is closely associated with alterations in the GM. As a complex and diverse ecosystem, the GM resides symbiotically within the gastrointestinal tract and plays critical roles in host immunity, metabolism, and the maintenance of intestinal barrier homeostasis (Riccio and Rossano, 2020). During episodes of diarrhea, however, disruption of this microbial ecosystem leads to GM dysbiosis, which impairs metabolite production and immune responses, thereby compromising intestinal barrier function (Rengarajan et al., 2020; Shi Z. et al., 2023). Deficiency of beneficial bacteria and overgrowth of certain pathogens (e.g., *E. coli* and *Shigella*) is one of the important pathogenic mechanisms of diarrhea (Baker and The, 2018; Khan et al., 2022).

Diarrhea can alter the composition of the GM, and in turn, the application of probiotics, FMT or bacteriophage can directly or indirectly influence the GM and the therapeutic outcome of diarrhea. Currently, probiotics, FMT and bacteriophage have demonstrated considerable anti-diarrheal potential, which can regulate the immune response and enhance intestinal barrier function by regulating the diversity and composition of GM and the content of metabolites, and effectively improving diarrhea symptoms (Pilla and Suchodolski, 2019; Sánchez et al., 2017). Notably, the use of probiotics for treating diarrhea is well-documented. These interventions broadly fall into three categories: single-strain preparations, multi-strain mixtures, or probiotics used in conjunction with conventional therapy. Probiotic supplementation not only helps prevent the occurrence of diarrhea but also enhances overall therapeutic efficacy and clinical cure rates, while shortening the duration of symptoms. Importantly, probiotic interventions are associated with a low incidence of adverse reactions (Liu et al., 2022; Steyer et al., 2022). Commonly used probiotics including *Lactobacillus* and *Bifidobacterium*, etc. Probiotics can gain a competitive advantage by altering the intestinal environment, e.g., inhibiting the growth of pathogenic bacteria through the competitive exclusion of intestinal binding sites; probiotics can up-regulate the synthesis of TJ proteins and then protect the intestinal barrier; and they can inhibit the production of pro-inflammatory cytokines to regulate intestinal immune function (Du et al., 2023). In addition, the application of FMT for the treatment of diarrhea has gained increasing attention in recent years owing to its favorable safety profile. The infusion of fecal material from healthy donors can help restore the GM of diarrhea patients to a state resembling that of the donor, thereby alleviating diarrheal symptoms (Li et al., 2019; Zheng et al., 2020). Currently, most studies have documented and summarized the phenomenon of healing in patients with diarrhea after treatment with FMT, such as clinical cure rate and duration of action (Lee et al., 2018; Pereira et al., 2018). Other investigations have explored alterations in the composition and structure of the GM post-FMT, as well as its effects on intestinal immune-inflammatory responses and barrier function, to elucidate the mechanisms underlying the alleviation of diarrhea (Li, 2020; Tian et al., 2022). Compared to probiotics and FMT, phage therapy demonstrates unique application value in the treatment of diarrhea due to its highly specific antibacterial effects (Chen X. et al., 2023). Phages can precisely recognize and lyse specific pathogenic bacteria (such as diarrheagenic *E. coli* and *Salmonella*), while preserving the stability of beneficial bacterial communities, thereby enabling precise modulation of the GM (Battistelli et al., 2024; Cui et al., 2022). Research has demonstrated that phage therapy can effectively alleviate symptoms of bacterial diarrhea, reduce levels of inflammatory cytokines, and promote the repair of the intestinal mucosal barrier (Dong et al., 2024). However, despite these results indicating the significant potential of phages in combating diarrhea, their clinical application still faces a critical translational challenge: the relatively poor stability of phages in the hostile gastrointestinal environment. Gastric acid and digestive enzymes can readily cause rapid denaturation of phage particles, thereby compromising therapeutic efficacy (Nobrega et al., 2016). Current research primarily focuses on the clearance of pathogens by phages and preliminary evaluation of clinical efficacy, while studies on post-treatment changes in gut microbiota diversity, metabolite profiles, and immune mechanisms remain relatively limited. Future efforts should involve more rigorous randomized controlled trials, along with optimization of phage characterization, sensitivity detection, and dosing regimens.

A review of the relationship between GM and disease reveals that GM dysbiosis is a critical factor in the pathogenesis of diarrhea. Alterations in the GM can lead to abnormal levels of microbial metabolites, such as SCFAs and BAs. These changes in the GM and its metabolites may further modulate immune cell functions and inflammatory factor levels, ultimately contributing to the onset of diarrhea. Although probiotics, FMT, and bacteriophage have been widely used in the treatment of diarrhea, and their efficacy and safety have encouraged the development of therapeutic approaches for gastrointestinal and other systemic diseases, there are still some issues that need to be addressed. Firstly, there is a scarcity of large-scale clinical trials, and secondly, the underlying mechanisms have not been sufficiently clarified through basic experimental research. Therefore, probiotic and bacteriophage interventions, as well as fecal microbiota transplantation, as safe and effective anti-diarrheal treatment strategies still need to go through a long journey.

## 5 Conclusion

Overall, GM alterations represent a crucial factor in diarrhea pathogenesis and a key target for its treatment. Diarrhea incidence has been closely linked to elevated levels of Proteobacteria and reduced Firmicutes; thus, targeted GM modulation aids in alleviating diarrhea symptoms. Currently, based on the principle of alleviating gut microbiota disorders, the use of probiotics (such as *Lactobacillus* and *Bifidobacterium*), FMT, and bacteriophages has been demonstrated to have definite effects on diarrhea. However, substantial clinical and basic research is still required to elucidate the optimal selection of these interventions, such as screening probiotic strains, FMT donors, and bacteriophages with superior pathogen-targeting advantages, as well as to investigate their long-term safety and efficacy in the treatment of diarrhea. Encouragingly, advances in multi-omics technologies have greatly facilitated investigations into diarrhea treatment mechanisms. Future studies should actively employ diverse research approaches to explore the potential mechanisms of different interventions in various diarrheal diseases and other related conditions, thereby providing data support for clinical diarrhea management and a foundation for the development of novel anti-diarrheal agents.
